# Synergetic effect of yeast cell-surface expression of cellulase and expansin-like protein on direct ethanol production from cellulose

**DOI:** 10.1186/1475-2859-12-66

**Published:** 2013-07-08

**Authors:** Yuki Nakatani, Ryosuke Yamada, Chiaki Ogino, Akihiko Kondo

**Affiliations:** 1 Department of Chemical Science and Engineering, Graduate School of Engineering, Kobe University, 1-1 Rokkodaicho, Nada-ku, Hyogo, 657-8501, Japan; 2 Organization of Advanced Science and Technology, Kobe University, 1-1 Rokkodaicho, Nada-ku, Hyogo, 657-8501, Japan

**Keywords:** Bioethanol, Cellulase, Cellulose, Cell-surface display, Expansin-like protein, Swollenin, Yeast

## Abstract

**Background:**

Numerous studies have examined the direct fermentation of cellulosic materials by cellulase-expressing yeast; however, ethanol productivity in these systems has not yet reached an industrial level. Certain microorganisms, such as the cellulolytic fungus *Trichoderma reesei*, produce expansin-like proteins, which have a cellulose-loosening effect that may increase the breakdown of cellulose. Here, to improve the direct conversion of cellulose to ethanol, yeast *Saccharomyces cerevisiae* co-displaying cellulase and expansin-like protein on the cell surface were constructed and examined for direct ethanol fermentation performance.

**Results:**

The cellulase and expansin-like protein co-expressing strain showed 246 mU/g-wet cell of phosphoric acid swollen cellulose (PASC) degradation activity, which corresponded to 2.9-fold higher activity than that of a cellulase-expressing strain. This result clearly demonstrated that yeast cell-surface expressed cellulase and expansin-like protein act synergistically to breakdown cellulose. In fermentation experiments examining direct ethanol production from PASC, the cellulase and expansin-like protein co-expressing strain produced 3.4 g/L ethanol after 96 h of fermentation, a concentration that was 1.4-fold higher than that achieved by the cellulase-expressing strain (2.5 g/L).

**Conclusions:**

The PASC degradation and fermentation ability of an engineered yeast strain was markedly improved by co-expressing cellulase and expansin-like protein on the cell surface. To our knowledge, this is the first report to demonstrate the synergetic effect of co-expressing cellulase and expansin-like protein on a yeast cell surface, which may be a promising strategy for constructing direct ethanol fermenting yeast from cellulose.

## Background

Lignocellulosic biomass has attracted considerable recent attention as a renewable and abundant energy source because of the environmental problems associated with the combustion of fossil fuels. In particular, cellulose, which is the main component of lignocellulosic biomass, is a promising starting material for the sustainable production of chemicals and fuels, such as bioethanol [1]. However, because many fermenting microorganisms cannot assimilate cellulose directly, the enzymatic saccharification of cellulose is required for producing fermentable glucose.

The saccharification of cellulose requires the synergetic activity of three types of cellulases: cellobiohydrolase (CBH, EC 3.2.1.91), endoglucanase (EG, EC 3.2.1.4), and β-glucosidase (BGL, EC 3.2.1.21). As large amounts of cellulase enzymes are necessary for cellulose saccharification, endowing non-cellulolytic microorganisms with cellulolytic activity has been frequently studied as an approach to reduce the need for added cellulase, which increases costs [2-5]. For example, cellulase genes from various kinds of microorganisms have been expressed in the ethanol-producing yeast *Saccharomyces cerevisiae* with the aim of directly producing ethanol from cellulose [6-10]. In addition, yeast strains displaying cellulase on the cell surface have also been developed for improving the efficiency of direct ethanol production from cellulose [10]. Yamada et al. [11] reported direct ethanol production from phosphoric acid swollen cellulose (PASC) and pretreated rice straw by a yeast strain constructed using a novel expression optimization method to co-display *Trichoderma reesei* EGII and CBHII, and *Aspergillus aculeatus* BGL1 on the cell surface. Despite these research efforts, the efficiency of ethanol production from cellulose remains too low for industrial lignocellulosic ethanol production processes.

Due to the rigid structure of cellulose, various types of proteins other than cellulases are needed to accelerate cellulose degradation for efficient saccharification [12]. One such protein is called swollenin, which was first identified as a plant expansin-like proteins in the cellulolytic fungi *T. reesei* by Saloheimo et al. [13], who reported that swollenin could swell cotton fibers without producing detectable amounts of reducing sugars. Swollenin was later shown to be capable of weakening and disrupting hydrogen-bond networks in lignocellulose [14]. The loosening effect of swollenin makes lignocellulosic biomass more accessible and readily hydrolyzable by cellulase, thereby promoting the degradation of lignocellulose during fermentation [15,16]. Although the swollenin gene from *T. reesei* has been heterologously expressed in *S. cerevisiae*[13], *Kluyveromyces lactis*[17], and *Aspergillus niger*[13], no studies have examined the effect of the co-expression of cellulase and swollenin in a single organism on the direct assimilation of cellulose.

In the present study, to improve the cellulolytic activity of cellulase-displaying yeast for direct and efficient ethanol production from the fermentation of cellulose, we attempted to co-display cellulase and expansin-like protein on the cell surface of *S. cerevisiae*.

## Methods

### Strains and media

Table 1 summarizes the genetic properties of the strains and plasmids used in this study. *Escherichia coli* strain NovaBlue (Novagen, Madison, WI, USA) was used as a host for recombinant DNA manipulations. Cellulases and expansin-like proteins were expressed in *S. cerevisiae* strain MT8-1 [18]. The previously constructed cellulase surface displaying *S. cerevisiae* strain MT8-1/cocδBEC1 [19], which has 8 copies of EGII gene from *T. reesei*, 2 copies of CBHII gene from *T. reesei*, and 1 copy of BGL1 gene from *A. aculeatus*, was also used for co-expression of cellulase and expansin-like protein.

**Table 1:** Strains and plasmid used in this study

**Strains and plasmids**	**Relevant features**	**Reference**
*E. coli* strain		
Novablue	*endA1 hsdR17(r*_*K12*_^*-*^*m*_*K12*_^*+*^*) supE44 thi-I gyrA96 relA1 lac recA1/F’[proAB*^*+*^*lacI*^*q*^ ZΔM15::Tn10(Tet^r^)]	Novagen
*S. cerevisiae* yeast strains		
MT8-1	*MATa ade leu2 his3 ura3 trp1*	[18]
MT8-1/cocδBEC1	*MATa ade leu2 his3 ura3*, expressing β-glucosidase, endoglucanase and cellobiohydrolase genes on its cell surface	[19]
MT8-1/δSWO	*MATa ade leu2 his3 trp1*, expressing SWOI genes from *T. reesei* on its cell surface	This study
MT8-1/δELPAO	*MATa ade leu2 his3 trp1*, expressing AoelpI genes from *A. oryzae* on its cell surface	This study
MT8-1/cocδBEC1/δSWO	*MATa ade leu2 his3*, expressing β-glucosidase, endoglucanase, cellobiohydrolase, and SWOI genes on its cell surface	This study
MT8-1/cocδBEC1/δELPAO	*MATa ade leu2 his3*, expressing β-glucosidase, endoglucanase, cellobiohydrolase, and AoelpI genes on its cell surface	This study
Plasmids		
pδU-PGAGSWO	URA3, expression of SWOI by δ-integration	This study
pδU-PGAGELPAO	URA3, expression of AoelpI by δ-integration	This study

*E. coli* transformants were grown in Luria-Bertani medium (10 g/L tryptone, 5 g/L yeast extract, and 5 g/L NaCl [Nacalai Tesque, Kyoto, Japan]) supplemented with 100 μg/mL ampicillin. Yeast transformants were screened using synthetic dextrose (SD) medium (6.7 g/L yeast nitrogen base without amino acids (Difco Laboratories, Detroit, MI, USA) and 20 g/L glucose [Nacalai Tesque]) or synthetic PASC (SPASC) medium (6.7 g/L yeast nitrogen base without amino acids and 10 g/L PASC) supplemented with appropriate amino acids and nucleic acids. PASC was prepared from Avicel PH-101 (Fluka Chemie GmbH, Buchs, Switzerland) as amorphous cellulose [3].

Yeast cells were aerobically cultured in 1-liter flasks containing 500 mL yeast/peptone/dextrose (YPD) medium (10 g/L yeast extract, 20 g/L Bacto-peptone (Difco Laboratories), and 20 g/L glucose) with rotary shaking at 150 rpm and 30°C. Ethanol fermentation was performed using YP medium (10 g/L yeast extract and 20 g/L Bacto-peptone Peptone) supplemented with 20 g/L PASC.

### Plasmid construction

The universal δ-integrative plasmid for cell-surface expression was constructed as follows. A DNA fragment encoding the promoter sequence of the *S. cerevisiae PGK1* gene, secretion signal sequence of SAG1, and the 3′ half of the α-agglutinin gene, including the terminator sequence, was excised from plasmid PGK406 AG [20] by XhoI/NotI digestion, and then inserted into the SalI/NotI site of plasmid pδU [21]. The resultant plasmid was named pδUPGSecAG.

The SWOI or AoelpI cell-surface expression plasmids were constructed as follows. The genes encoding the expansin-like proteins SWOI from *T. reesei*[13] and AoelpI from *A. oryzae*, which was identified by sequence similarity to an expansin-like protein of *A. niger* (CAK48166), were amplified from cDNA of *T. reesei* and *A. oryzae* with the primers SWO(F) and SWO(R), and Aoelp(F) and Aoelp(R), respectively. Then amplified fragments were digested with BglII/SalI restriction enzymes and inserted into the BglII/SalI site of plasmid pδUPGSecAG, generating plasmids pδUPGAGSWO and pδUPGAGELPAO, respectively.

### Yeast transformation

A *S. cerevisiae* strain expressing the expansin-like proteins SWOI or AoelpI on the cell surface was constructed by the δ-integration method, as described previously [19]. The δ-integrative plasmids pδUPGAGSWO and pδUPGAGELPAO, which allow surface expression of SWOI and AoelpI, respectively, were transformed into MT8-1, generating strains MT8-1/δSWO and MT8-1/δPAO, respectively.

A *S. cerevisiae* strain co-expressing the expansin-like protein SWOI or AoelpI and cellulases EG, CBH, and BGL on the cell surface was constructed by δ-integration of pδUPGAGSWO or pδUPGAGELPAO into MT8-1/cocδBEC1, generating strains MT8-1/cocδBEC1/δSWO and MT8-1/cocδBEC1/δPAO, respectively.

### PASC degradation activity of cellulolytic yeast strain

PASC degradation experiments were carried out to evaluate the synergism between the cellulase-expressing and expansin-like protein-expressing yeast strains, or PASC degradation activity of cellulase and expansin-like protein co-expressing strain. Each strain was cultivated in YPD medium for 48 h at 30°C (initial optical density (OD) at 600 nm was 0.05), collected by centrifugation at 3,000 × g for 5 min at 4°C, and then washed twice with distilled water. After weighing the cell pellet (the estimated dry-cell weight for all strains was approximately 0.15 times that of the wet-cell weight), the washed cells of each strain were added at a final concentration of 50 g wet cell/L in the case of single-strain or 25 g-wet cell/L in the case of mix of cellulase-expressing strains (final concentration of 50 g wet cell/L in all reactions) to 5 g/L PASC with 50 mmol/L sodium citrate buffer (pH 5.0) and 100 mmol/L methyl glyoxal (Nacalai Tesque), which was added to prevent assimilation of the produced glucose by yeast cells [11]. The hydrolysis reaction was performed at 50°C for 2 h with shaking at 150 rpm, and the supernatant was then collected by centrifugation at 10,000 × g for 5 min at 4°C to remove cells and PASC residue. The glucose concentration of the supernatant was measured by the Glucose CII test (Wako Pure Chemical Industries, Ltd., Osaka, Japan). One unit of PASC degradation activity was defined as the amount of enzyme producing 1 μmol/min glucose at 50°C, pH 5.0.

### Quantification of cellulase-encoding genes transcription by real-time RT-PCR

The transcription level of each cellulase-encoding gene was quantified by real-time reverse transcription (RT)-PCR using a Stratagene MX3000P qPCR system and Thunderbird SYBR qPCR Mix (Toyobo, Osaka, Japan). Total RNA was isolated from yeast cells cultivated in YPD medium for 48 h at 30°C using a RiboPure Yeast Kit (Ambion, Austin, TX, USA), and was then used for cDNA synthesis using a ReverTra Ace qPCR RT Kit (Toyobo). The synthesized cDNA was used as a template for real-time RT-PCR, which was performed with three sets of PCR primers: (BGL761(F) and BGL858(R), EGII968(F) and EGII1043(R), CBHII387(F) and CBHII455(R), and AoelpI567(F) and AoelpI637(R)) (Table 2). Transcription levels of the three cellulase genes and the AoelpI gene were normalized to the housekeeping gene *PGK1* using a standard curve method.

**Table 2:** Polymerase chain reaction primers used in this study

**Primer**	**Sequence**
SWO(F)	5’-ATATGTCGACCAGAATTGCGCAGCATTATTTG-3’
SWO(R)	5’-ATATAGATCTATTCTGGCTAAACTGCACACCA-3’
AOELP(F)	5’-ATATGTCGACGCAGACATATGCCGTTACCTTG-3’
AOELP(R)	5’-ATATAGATCTCCGCCGGCGGGCTGGATCTCTT-3’
BGL761(F)	5’-CTTCCAGGGCTTTGTGATGTC-3’
BGL858(R)	5’-AGGTGATATCGCCAGGCATT-3’
EGII968(F)	5’-GAACAATCGCCAGGCTATCCT-3’
EGII1043(R)	5’-TTGCTGGCACATGTCTTGTATG-3’
CBHII387(F)	5’-GGTTCCCTCTTTTATGTGGCTAGA-3’
CBHII455(R)	5’-ATGTCGGCCAAGGTTTGCT-3’
AoelpI567(F)	5’-TGGCCCTGGTTATGAAACAGA-3’
AoelpI637(R)	5’-CGGTAGTAGCAGGGTAAGCTATTCC-3’

### Ethanol fermentation from PASC

Yeast cells were precultured aerobically in YPD medium at 30°C for 72 h, harvested by centrifugation at 1,000 × g for 5 min, and then washed twice with distilled water. The wet-cell weight was then determined by harvesting the washed cells by centrifugation at 3,000 × g for 5 min. The cells were then resuspended in 20 mL YP medium containing 20 g/L PASC at an initial cell concentration adjusted to 200 g wet cell/L.

Ethanol fermentation was performed at 37°C for 96 h with mild agitation in 100-ml closed bottles equipped with a siliconized tube and CO_2_ outlet valve (Sanplatec Corp., Osaka, Japan). Ethanol concentration was determined using a gas chromatograph (model GC-2010; Shimadzu, Kyoto, Japan) equipped with a flame ionization detector and a Durabond Free Fatty Acid Phase (DB-FFAP) column (60 m × 0.25 mm internal diameter, 0.5-μm film thickness; Agilent Technologies, Palo Alto, CA, USA) using helium as the carrier gas. The injection volume and split ratio was adjusted to 1 μL and 1:50, respectively. The column temperature was programmed to increase from 40 to 170°C with a linear gradient of 10°C/min.

## Results

### Synergism between cellulase-expressing and expansin-like protein-expressing yeast strains

**Figure 1:**
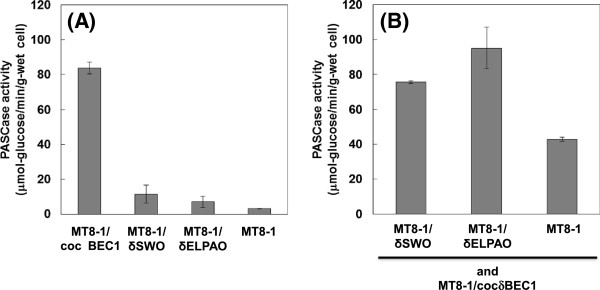
**Synergism between cellulase-and expansin-like protein-expressing yeast strains. (A)** PASCase activity of single-strain. **(B)** PASCase activity of the indicated mix of cellulase-expressing strain. Data are averages from three independent experiments (error bars represent SE).

To confirm the synergetic effect of yeast cell surface-displayed expansin-like proteins (SWOI and AoelpI) and cellulases (EGII, CBHII, and BGL) on cellulose degradation, the PASC degradation ability of a mix of the cellulase-expressing strain MT8-1/cocδBEC1 and a yeast strain MT8-1/δSWO or MT8-1/δPAO displaying the expansin-like protein SWOI or Aoep1 was evaluated (Figure 1). As shown in Figure 1A, the amount of glucose produced from PASC by the yeast strains displaying expansin-like proteins was similar to that of wild-type strain MT8-1. However, the mix of MT8-1/cocδBEC1 and expansin-like protein-displaying strain produced more glucose than that of the mix of MT8-1/cocδBEC1 and MT8-1 (Figure 1B). The mix of Aoelp-expressing strain and MT8-1/cocδBEC1 produced the highest amount of glucose (95.1 μmol-glucose/min/g-wet cell), representing a 2.2-fold increase over the amount generated by the mix of MT8-1 and MT8-1/cocδBEC1 (42.8μmol-glucose/min/g-wet cell). These results suggested that yeast cell-surface expressed expansin-like protein and cellulase acted synergistically in the degradation of cellulose.

### Yeast cell surface co-expression of cellulase and expansin-like protein

**Figure 2:**
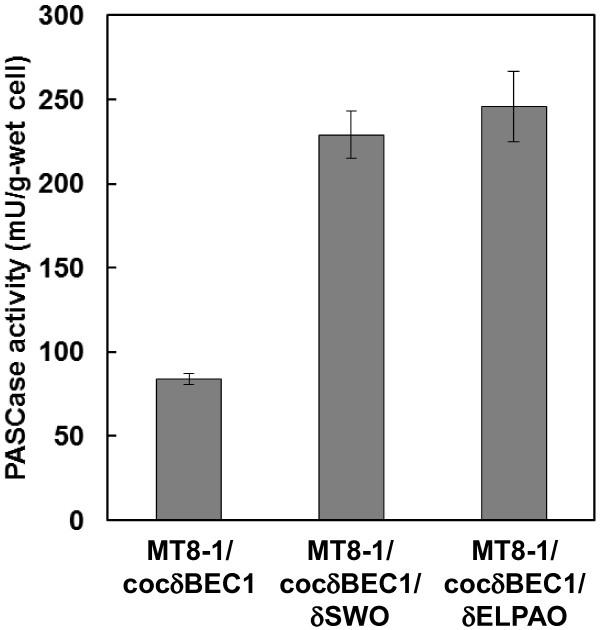
**PASCase activity of cellulase and expansin-like protein co-expressing strain.** Data are averages from three independent experiments (error bars represent SE).

To confirm the synergistic effect of cell-surface expressed cellulase and expansin-like protein on the degradation of PASC, the PASCase activity of a cellulase and expansin-like protein co-expressing strain was examined (Figure 2). The PASCase activities of strains co-expressing AoelpI and cellulase, and SWOI and cellulase were 246 and 229 mU/g-wet cell, respectively, which corresponded to 2.9- and 2.3-fold higher PASCase activity, respectively, than the cellulase-expressing strain MT8-1/cocδBEC1.

### Quantification of cellulase-encoding genes transcription by real-time RT-PCR

**Figure 3:**
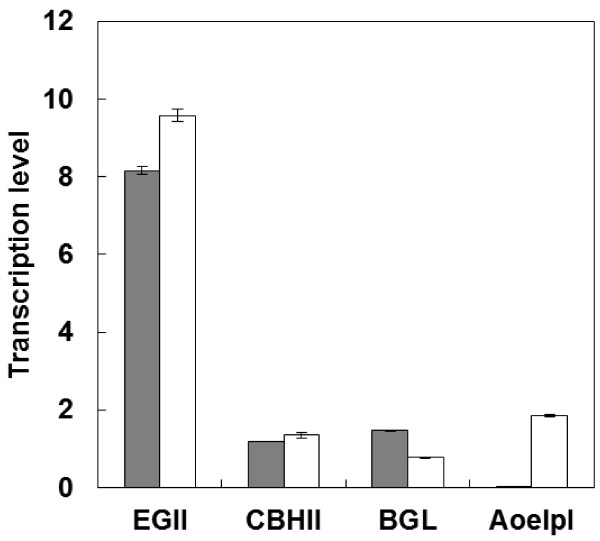
**Transcription anscription levels of cellulase and expansin-like protein genes.** Gray bars, MT8-1/cocδBEC1; white bars, MT8-1/cocδBEC1/δELPAO. Data are averages from three independent experiments (error bars represent SE).

To evaluate the effect of AoelpI expression on cellulase genes expression level, the transcription level of cellulase-encoding genes in the cellulase-expressing strain MT8-1/cocδBEC1 and AoelpI and cellulase co-expressing strain MT8-1/cocδBEC1/δELPAO was evaluated by real-time RT-PCR (Figure 3). The transcriptional levels of the EGII, CBHII, and BGL genes in the AoelpI and cellulase co-expressing strain (9.6, 1.4, and 0.8, respectively) were similar to those in the cellulase-expressing strain (8.2, 1.2, and 1.5, respectively). However, AoelpI transcription was detected only in the AoelpI and cellulase co-expressing strain.

### Ethanol production from PASC by a yeast strain co-expressing cellulose and swollenin

**Figure 4:**
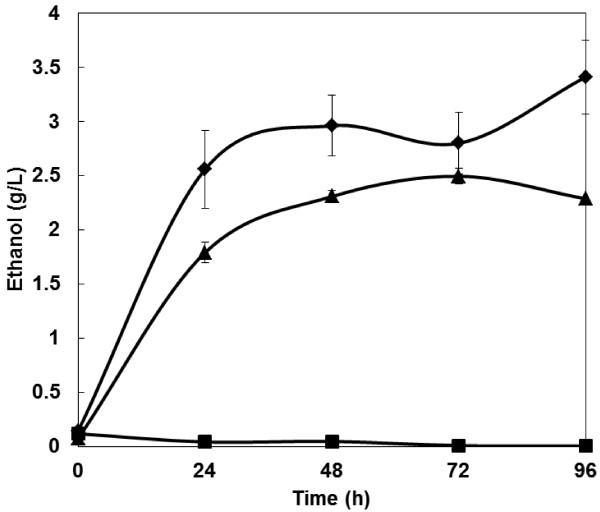
**Time course of ethanol production from PASC by an engineered yeast strain.** Squares, MT8-1; triangles, MT8-1/cocδBEC1; and diamonds, MT8-1/cocδBEC1/δELPAO. Data are averages from three independent experiments (error bars represent SE).

The synergistic effect of AoelpI and cellulase co-expression on direct ethanol production from cellulose was investigated using PASC as a fermentation substrate (Figure 4). Compared to the cellulase-expressing strain, which produced 2.5 g/L of ethanol during a 96-h fermentation, the AoelpI and cellulase co-expressing strain produced 3.4 g/L of ethanol during the same period, representing a 1.4-fold increase.

## Discussion

In this study, the synergetic effect of yeast cell-surface expressed cellulase and expansin-like protein on the degradation of PASC was evaluated. To improve cellulose degradation efficiency and direct ethanol fermentation performance from cellulose, a yeast strain co-displaying expansin-like protein and cellulase was constructed and evaluated for its direct ethanol fermentation ability from PASC. Using this approach, the PASC degradation and fermentation ability was markedly improved.

Saloheimo et al. [13] first reported that the swollenin protein from the cellulolytic fungus *T. reesei* was capable of disrupting the structure of cellulose without producing detectable amounts of reducing sugars. Other expansin-like proteins were subsequently isolated from other types of microorganisms, including *A. fumigatus*[16], *T. asperellum*[14], *T. pseudokoningii*[22], and *Bacillus subtilis*[23]. Here, expansin-like proteins from *T. reesei* (SWOI) and *A. oryzae* (AoelpI) expressed on the *S. cerevisiae* cell surface had synergetic effects on PASC degradation (Figure 1). Based on the findings of co-expression of cellulase and expansin-like protein (Figure 2), cell-surface expressed AoelpI and SWOI had the efficient synergetic effect with cellulase for degradation of PASC. It was reported that the secreted cellulases and expansin-like protein act synergistically [14,16,22]. In this study, these proteins have positive synergistic effect even on cell surface. At present, because the role of expansin-like protein for degradation of cellulose has not been reveled in detail yet, the mechanism of synergetic reaction with cellulase is unclear. Thus, it would be expected that the mechanism is figured out and expansin-like protein is used for efficient degradation of cellulose in future works.

The co-expression of expansin-like protein AoelpI or SWOI and three types of cellulase enzymes on the cell surface of *S. cerevisiae* resulted in higher PASCase activity than the strain expressing only cellulase (Figure 2). Because the transcription levels of the cellulase genes in the expansin-like proteins and cellulase co-expressing strain were similar with those in the cellulase-expressing strain, it is likely that expansin-like proteins were successfully expressed on the cell surface. To our knowledge, this is the first report concerning the functional expression of an expansin-like protein on a yeast cell surface.

For efficient saccharification of PASC, the optimal expression ratio of the three types of cellulases is necessary [19]. To optimize the expression ratio of multiple target genes in *S. cerevisiae*, the cocktail δ-integration method was developed in a previous study [19]. Using this method, an expansin-like protein and cellulase co-expressing *S. cerevisiae* strain was constructed in which the transcription level of EG was significantly higher than that of CBHII, BGL, and AoelpI (Figure 3). For degradation of low-crystallinity substrates such as PASC, EG activity is the most important of the three types of cellulases [19]. Thus, the cellulase expression ratio in the expansin-like protein and cellulase co-expressing strain was successfully optimized for cellulose fermentation. This result is in good agreement with a previous report [19]. Notably, the AoelpI transcription level in the expansin-like protein and cellulase co-expressing strain was relatively low, suggesting that AoelpI may act synergistically with cellulase even at low expression levels. Together, these findings suggest that the cocktail δ-integration method is a promising strategy for constructing multi-gene expressing yeast strains.

The expansin-like protein and cellulase co-expressing strain showed higher ethanol productivity from PASC than the cellulase-expressing strain (Figure 4). The higher PASCase activity of the co-expressing strain would partly explain this result. Although the loosening effect for crystalline cellulose such as Avicel has been well studied, that for amorphous cellulose such as PASC used in this study has been little known. Chen et al. [16] reported that swollenin-like protein from *Aspergillus fumigatus* showed weak endoglucanase activity for amorphous cellulose CMC. The expansin-like proteins used in this study may also have weak endoglucanase activity (Figure 1). However, it is difficult to explain the improvement of PASC degradation activity and ethanol production rate with such a weak endoglucanase activity. Thus, there might be the unknown effects for degradation of amorphous cellulose by expansin-like protein. It is expected that the mechanisms of loosening effect of expansin-like protein is revealed in detail in future works. At least, co-expression of cellulase and expansin-like protein on the yeast cell surface is expected to improve ethanol productivity from cellulose.

## Conclusions

The PASC degradation and fermentation ability of an engineered *S. cerevisiae* strain was improved by co-expressing cellulase and expansin-like protein on the cell surface. To our knowledge, this is the first report to demonstrate the synergetic effect of co-expressing cellulase and expansin-like protein on a yeast cell surface. Because this study is the proof of concept, the ethanol productivity from cellulose was low compared to the levels typically achieved in industrial ethanol production processes. Moreover, the engineered lab strain might not have enough robustness for industrial application. Thus, strain improvement and fermentation engineering strategy such as polyploidization and fed-batch cultivation should be required for industrial application [21,24]. However, because the cell-surface expression of expansin-like protein improved not only the ethanol production rate from cellulose, but also the ethanol yield with relatively low expression level, co-expression of cellulase and expansin-like protein on the cell surface of yeast may be a promising strategy for constructing yeast strains capable of directly fermenting ethanol from cellulose.

## Competing interests

The authors declare that they have no competing interests.

## Authors’ contributions

YN designed and performed the experiments. YN and RY wrote the paper. CO and AK commented and supervised on the manuscript. All the authors approved the final manuscript.
